# The rat placental renin-angiotensin system - a gestational gene expression study

**DOI:** 10.1186/s12958-015-0088-y

**Published:** 2015-08-12

**Authors:** Kanchan Vaswani, Hsiu-Wen Chan, Pali Verma, Marloes Dekker Nitert, Hassendrini N. Peiris, Ryan J. Wood-Bradley, James A. Armitage, Gregory E. Rice, Murray D. Mitchell

**Affiliations:** Centre for Clinical Diagnostics, University of Queensland Centre for Clinical Research Royal Brisbane and Women’s Hospital Campus, Building 71/918, Royal Brisbane Hospital, Herston, QLD 4029 Australia; Department of Anatomy & Developmental Biology Monash University, Clayton, VIC 3800 Australia; School of Medicine (Optometry), Deakin University, Pigdons Road, Waurn Ponds, VIC 3800 Australia

**Keywords:** Placenta, Renin-Angiotensin System, Microarray, Gestation

## Abstract

**Background:**

The placenta is an essential organ that provides nutrients and oxygen to the developing fetus and removes toxic waste products from the fetal circulation. Maintaining placental blood osmotic pressure and blood flow is crucial for viable offspring. The renin-angiotensin system (RAS) in the placenta is a key player in the regulation of maternal-fetal blood flow during pregnancy. Therefore, the aim of this study was to determine if RAS genes are differentially expressed in mid to late gestation in rat placenta.

**Methods:**

Whole placental tissue samples from pregnant Sprague Dawley rats at embryonic (E) days 14.25, 15.25, 17.25 and 20 (*n* = 6 for each gestational age) were used for genome-wide gene expression by microarray. RAS genes with expression differences of >2 fold were further analyzed. Quantitative Real-Time PCR (qPCR) was performed on independent samples to confirm and validate microarray data. Immunohistochemisty and Western blotting were performed on a differentially expressed novel RAS pathway gene (ANPEP).

**Results:**

Six out of 17 genes of the RAS pathway were differentially expressed at different gestational ages. Gene expression of four genes (Angiotensin converting enzyme (*Ace*), angiotensin converting enzyme 2 (*Ace2*), membrane metalloendopeptidase (*Mme*) and angiotensin II receptor 1A *(Agtr1a)*) were significantly upregulated at E20 whereas two others (Thimet oligopeptidase 1 (*Thop1*) and Alanyl aminopeptidase (*Anpep)*) were downregulated at E20 prior to the onset of labour. These changes were confirmed by qPCR. Western blots revealed no overall differences in ANPEP protein expression in the placentae. Immunohistochemical studies, however, indicated that the localization of ANPEP differed at E17.25 and E20 as ANPEP localization in the giant trophoblast cell of the junctional zone was no longer detectable at E20.

**Conclusions:**

The current study investigated the expression of members of the RAS pathway in rat placentae and observed significantly altered expression of 6 RAS genes at 4 gestational ages. These findings present the need for further comprehensive investigation of RAS genes in normal and complicated pregnancies.

**Electronic supplementary material:**

The online version of this article (doi:10.1186/s12958-015-0088-y) contains supplementary material, which is available to authorized users.

## Background

The Renin-Angiotensin System (RAS) regulates sodium and water homeostasis to maintain blood pressure and fluid balance in all mammals [1, 2]. The complex integration of actions of circulating maternal RAS in pregnancy plays a key role in pregnancy outcome and maternal health. In human pregnancy, the maternal and fetal circulating RAS interact with various tissue RASs (ovarian, intrauterine, and intrarenal) [3]. The intrauterine/placental RAS is one of the major extrarenal RAS in pregnancy [4], regulating maternal-fetal blood flow and the uteroplacental blood circulation [1, 5]. Other uteroplacental RAS functions may include endometrial regeneration, decidualisation, implantation, placentation, uterine contraction, prostaglandin synthesis, and estradiol 17 beta secretion [6, 7]. Because its functions are widespread, dysfunction of the RAS system can lead to complications such as preeclampsia. Placental RAS is itself regulated by several hormones including estrogen and progesterone [8].

The RAS pathway begins with the biosynthesis of active enzyme, renin, from its 47 kDa precursor, prorenin [9]. Renin cleaves angiotensinogen (AGT) to form angiotensin, AngI [10]. AngI is then converted to AngII by angiotensin-converting enzyme (ACE) [11, 12]. AngII binds two endogenous receptors: angiotensin II type 1 and 2 receptors (AT1 and AT2) encoded by genes *Agtr1* and *Agtr2* respectively [2, 4, 13–15]. *Agtr1* has two subtypes *Agtr1a* and *Agtr1b*. AngII is the primary product of RAS and its activity is terminated by its conversion to Ang 1–7 by MME, membrane metalloendopeptidase [6]. In the RAS pathway, the majority of AngII functions, which include vasoconstriction, vascular permeability and peripheral vascular resistance, stimulation of aldosterone synthesis, angiogenesis and cell growth, are mediated by AT1 [6, 15–17]. In contrast, AT2 is involved in apoptosis, reduction of endothelial cell growth, cell migration, vasodilation, and reduction of vascular injury [2]. AT2 has been hypothesised to antagonize the effects of AT1 [9, 18], and hence a distorted balance in the expression of these proteins may be detrimental to pregnancy outcomes. Placental RAS maybe involved in preeclampsia with raised AT1 expression [2]. In human placenta and placental cell lines, the genes involved in the RAS pathway have been detected in both early and late gestation leading to the speculation that it is critical throughout pregnancy [11]. As there is a paucity of data to demonstrate changes to RAS factors change as pregnancy progresses and in complications of pregnancy. Further investigation are required to determine the importance of RAS due to changes in expression and the functional changes associated.

RAS components have been shown to be localised to different regions and cell types of the placenta. AT1 has been mainly localised to human placental syncytiotrophoblasts [2]. The vasodilator ACE2 [1] has been detected in endothelium and *syncitium (i.e. multinucleated cells)* of labyrinth,, subplacenta (highly vascularised region, part of chorion found between placental disc and basal decidua) and giant cells, in guinea pigs [19]. In late pregnancy, ACE2 is present in the decidual layer and vascular smooth muscles of arteries modulating placental blood flow, regulating vascular remodeling, and potentiating trophoblast invasion and decidualisation earlier in pregnancy [19]. The vasodilator ACE2 has been detected in the endothelium, syncyotiotrophoblast, and cytotrophoblast, in human placenta tissue [20]. It is also present in the intravascular trophoblast and decidual layer and the vascular smooth muscle cells of the umbilical cord vessels [20]. In rats, the upregulation of ACE2 expression in the placenta and uterus contributed to a two-fold increase in total ACE2 activity [21]. Interestingly, although ACE2 expression was upregulated in the rat uterus, it was downregulated at the implantation site [22]. The maternal diet also modulates ACE2 expression; rat dams fed a low protein diet had reduced Ace2 mRNA expression in the placenta leading to fetal growth restriction [1]. Similar results were also reported in ACE2 knockout mice [23]. Overall, ACE2 is involved in modulating placental blood flow, regulating vascular remodeling, and potentiating trophoblast invasion and decidualisation. Moreover the formation of new blood vessels have been affected by the effects of hypoxia in Aminopeptidase-null mice, Aminopeptidase-N, ANPEP being another member of the RAS system [24].

In humans, trophoblast invasion, vascular remodelling and proper placentation are critical to determine pregnancy outcome. Poor vascular remodelling and trophoblast invasion can lead to complications such as preeclampsia. Preeclampsia is associated with short and long term consequences for both the mother and baby including preterm labour and still birth [14, 25, 26]. Alteration of RAS is involved in the pathogenesis of preeclampsia [27] indicating the essential role of RAS for the prevention of pregnancy complications. Gestational age-related studies of RAS throughout early, mid and late gestation are limited. Both systemic and uteroplacental RAS undergo dramatic changes during pregnancy. RAS components in human placenta are expressed from 6 weeks of gestation [12]. Recent studies on the human placenta compared the localisation and expression of RAS in early gestation vs late gestation [9, 18]. These studies showed that RAS plays a significant role in promoting trophoblast invasion and angiogenesis and is also linked to expression of PTGS2, Prostaglandin-Endoperoxide Synthase 2 (an enzyme potentially involved in the process of parturition).

RAS components have not been comprehensively studied in rat placenta from mid to late gestation. This study specifically aimed to determine gene expression changes of all 17 genes of the RAS pathway in rat placentae. Four time points were chosen as they cover the time period from mid gestation to the day prior to labour onset (E14.25, E15.25, E17.25 and E20). These investigations are apart of a larger gene expression study [28, 29]. The rat model allows the study of RAS compenents at time-points (mid to late gestation) difficult to be obtained from human and unlike in the mouse, placentation in the rat involves the deep invasion of trophoblast (*i.e.* a better model of placentation) [30].

## Methods

### Animals and diets

Animal experiments were performed at the Department of Anatomy and Developmental Biology, Monash University (Melbourne, Australia) with the approval of The School of Biomedical Sciences Animal Ethics Committee of the Monash University. Experiments were carried out in accordance with the National Health and Medical Research Council of Australia *“Australian Code of Practice for the Care and Use of Animals for Scientific Purposes”* (7th edition, 2004).

*Sprague Dawley* dams were used throughout the study. Rats were allowed to adapt to the animal house for one week. Throughout the study, animals were maintained on a diet of standard chow (19.5 % protein, 7 % total fat and 16 MJ/Kg digestible energy; Glen Forrest StockFeeders WA Rat and Mouse Chow) and water *ad libitum* prior to diet onset. Rats were maintained in a light-controlled environment (12 h light/dark cycle) throughout this study. Female rats were timed mated in a 3 h window with male *Sprague Dawley* rats. This was designated as Day 0 of pregnancy. The rationale of using a 3 h window for mating time is to reduce variability of gestational age among the offspring and to maximize the accuracy in staging of gestation. After mating, dams were housed individually.

### Tissue collection

The formation of the chorioallantoic rat placenta begins on gestational day 12, and therefore the study begins in mid-gestation. Pregnant dams were anaesthetized (Isoflurane Rhodia Australia P/L, VIC, Australia) and humanely killed at embryonic day (E) 14.25, 15.25, 17.25 or 20 (*n* = 6 per gestational age). Whole placentae were collected from the pregnant dams, weighed and then either snap frozen in liquid nitrogen or fixed in 4 % paraformaldehyde prior to processing for immunohistochemistry analysis. Frozen tissues were stored at −80 °C until processed and analysed.

### RNA isolation

Rat placental tissues were pulverized into a fine powder using liquid nitrogen and mortar and pestle. Total RNA was extracted from 30 mg of pulverized frozen placental tissue *n* = 6 placentae per gestational age group, using the AllPrep DNA/RNA Mini Kit (Qiagen) as per manufacturers’ instructions. An on-column Dnase1 treatment step was also carried out in the RNA isolation step to remove any remaining genomic DNA. Following extraction, total RNA was quantified via NanoDrop ND-1000 spectrophotometer (Thermo Scientific, DE, USA). RNA quality was determined with the Agilent 2100 Bioanalyzer (VIC, Australia). RNA samples that fulfilled the following criteria were selected for microarray analysis: (i) RIN > 8.5; (ii) 260/280 ratio >2; (iii) 260:230 ratio >2.2. All RNA Integrity Number values were greater than 8.7.

### Microarray analysis using Illumina rat ref arrays

The microarray analysis (cohort 1) was performed as previously published [28]. In short, 500 ng of total RNA was converted to double stranded cDNA and this was used to generate biotinylated cRNA probes using the Illumina TotalPrep RNA Amplification Kit. Biotin-labelled cRNA were then hybridized to Illumina RatRef-12 Expression BeadChip (San Diego, CA, USA). Slides were scanned on a BeadStation 500 System using Beadscan software version 3.5.31. No RNA samples were pooled in this analysis, each placental samples was analyzed independently. Samples were hybridized into wells at random. The Array experiment readout was deposited on ArrayExpress and includes gene expression data from microarray and high throughput sequencing studies (ArrayExpress Accession number E-MTAB-1987).

RAS genes from the KEGG Renin Angiotensin System pathway were studied for differential expression using SAM (Microarray Software, Stanford University) analyses, whereby differentially expressed placental genes (*i.e.* >2 fold expression change) were identified between each of the 4 gestational age groups.

### Quantitative real-time PCR validation

The RNA was reverse transcribed using the QuantiTect reverse transcription kit (Qiagen) using 1 μg of RNA per sample. qPCR (cohort 2) was used to confirm and validate the expression of *Ace*, *Ace2*, *Thop1*, *Mme* and *Anpep* in placentae that were different from those used for the microarray analysis. Primers unique for each target gene were designed by Primer-Blast (NCBI), and covered exon-exon junctions. The primer sequences are provided in Table 1.

**Table 1:** Primers used for the qPCR experiments for 6 RAS genes

Gene	Forward (5’-3’)	Reverse (5’-3’)
*Ace*	ATTGCAGCCGGGCAACTTTT	CGCATTCTCCTCCGTGATGT
*Ace2*	GAGCCCATATGCCGACCAAA	TCTGCCTCCCCAAAAGGAAC
*Mme*	CCGAAATGACCCAATGCTGC	TGACCAGCTGAATGGCTTCC
*Thop1*	ATGCTGGAGAACTGGGTGTG	GAAGAGACCTGCATTGGCCT
*Anpep*	AGATTGCCCTGCCTGACTTC	GTTGCCAAACCACTGATGGG
*Agtr1a*	GGATTCGTGGCTTGAGTCCT	TCGAAATCCACTTGACCTGGTG

### Immunohistochemistry

Paraffin sections (5 μm, *n* = 3 sections/gestational age) were deparaffinized in xylene and rehydrated to water through a graded alcohol series. Antigen retrieval was achieved by incubation in citrate buffer (10 mM), pH6 at 120 °C for 30 s and 90 °C for 10 min under higher pressure. Sections were blocked in MACH-1 Background snipper, provided in the MACH-1™ kit (Biocare Medical). Sections were incubated with rabbit anti-CD13 (ANPEP) polyclonal antibody (Abcam Cat no ab108310), prior to incubation with secondary antibodies conjuagated with HRP. They were then stained using DAB substrate, whereby positive staining was identified with a brown colour. Purified Rabbit IgG (Sigma Aldrich) was used as a negative isotype control. Sections were counterstained with Mayers hematoxylin and Leica mounting media was used to mount the coverslips. Slides were examined under a microscope (Olympus™).

### ANPEP western blots

Protein samples from all four gestational age groups (*n* = 6) were used to examine ANPEP protein expression by Western blotting. All reagents, unless otherwise stated, were purchased from Life Technologies for use in the NuPage Bis-Tris Electrophoresis System. Total protein extracted from rat placental tissue (30 μg) were separated in NuPage 4–12 % Bis-Tris Precast Gels and transferred to a polyvinyl difluoride (PVDF) membrane. Membranes were blocked for 1 h in blocking solution containing bovine serum albumin powder in Tris Buffered Saline with 0.001 % Tween 20 (Sigma Aldrich, Castle Hill, NSW, Australia). Membranes were incubated overnight at 4 °C with ANPEP (cd-13, Abcam cat no ab108310) (Santa Cruz Biotechnology) and β-Actin (Sigma-Aldrich)primary antibodies. Secondary antibodies were purchased from LI-COR™ Biociences. Odessey® LI-COR™ densitometry was used to determine changes in ANPEP (normalized to β-ACTIN protein) in the placental tissues.

### Statistical analysis

#### BioInformatic analyses and statistics

Microarray bioinformatic analysis was initially performed by using the Illumina Beadstudio and Significance Analysis of Microarry (SAM, Stanford University) software. Data were normalized by performing a probe-intensity transformation and normalization via the Lumi package, Bioconductor. Following normalization, differentially expressed placental genes (*i.e.* >2 fold expression, false discovery rate of <1 %) were identified using SAM and were subsequently analyzed using Web-based Gene Set Analysis Toolkit (WebGestalt, http://bioinfo.vanderbilt.edu/webgestalt/).

Genes were then assigned to their respective functional classes based on the Gene Ontology (GO) database; Renin-Angiotensin KEGG PATHWAY Analysis Differences in group means were assessed by post-hoc comparisons (Bonferroni tests).

For the qPCR and Western blots comparison of the four gestational groups, one-way ANOVA analyses and multiple comparisons were carried out. Correlation plots for both Microarray and qPCR on cohort 1 and 2 respectively were performed using Pearson’s Correlation (Additional file 1: Figure S1).

## Results

### Differentially expressed RAS genes

**Fig. 1:**
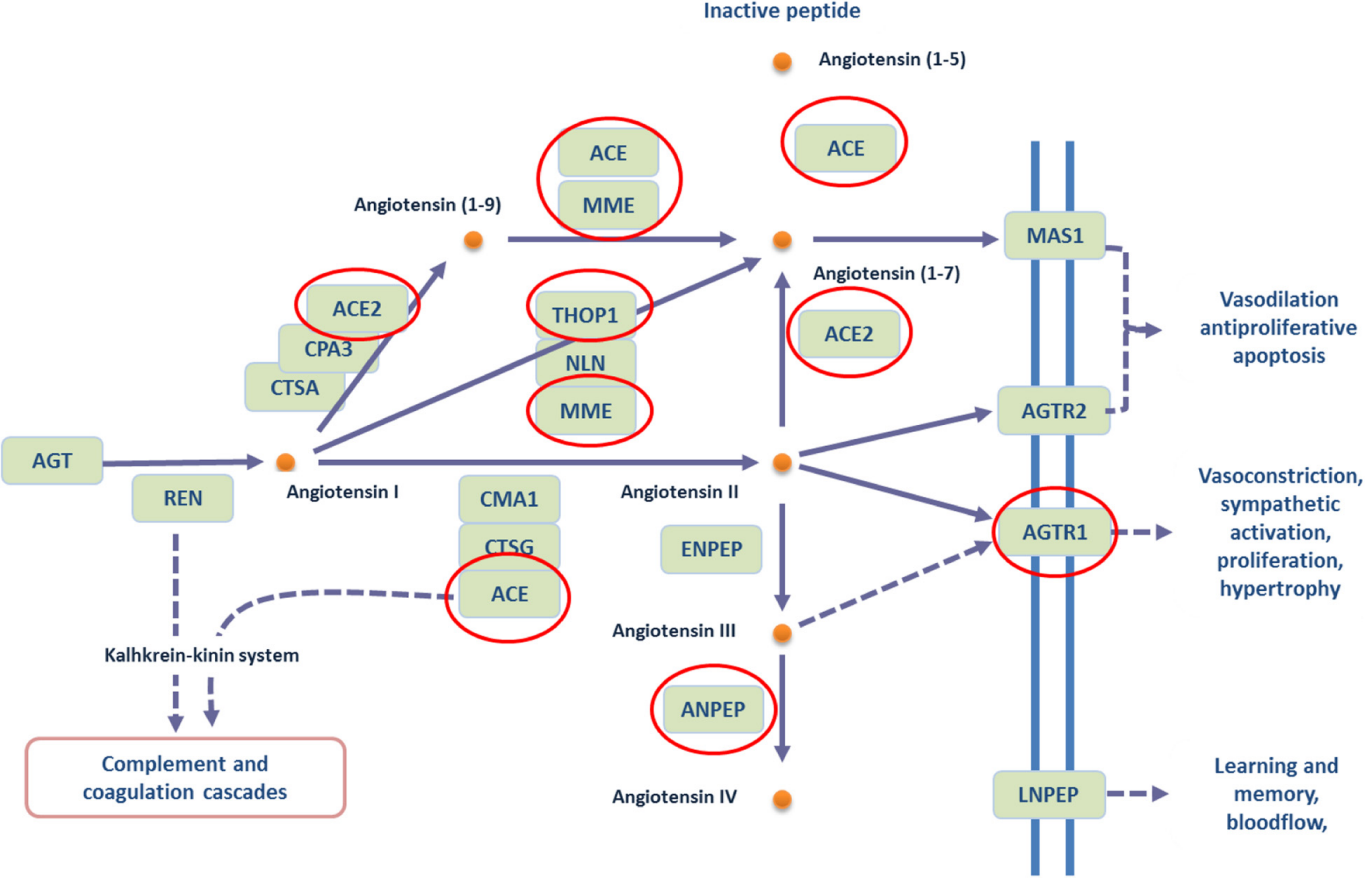
The Renin angiotensin system showing differentially expressed genes highlighted in red circles (adapted from the KEGG pathway). Solid lines and arrows (edges) denote direct relationships and dashed lines and arrows represent indirect relationships predicted and confirmed in the rat KEGG pathway. Arrows denote directional relationships and lines denote non-directional reported in the KEGG pathway. MME: membrane metalloendopeptidase; ANPEP: Alanyl aminopeptidase; MAS1: Mas-Related G Protein-Coupled Receptor (angiotensin 1–7 receptor); THOP1: Thimet oligopeptidase 1; ACE: Angiotensin converting enzyme; ACE2 angiotensin converting enzyme 2, AGTR1 and 2 Angiotensin II receptor 1 and 2

**Fig. 2:**
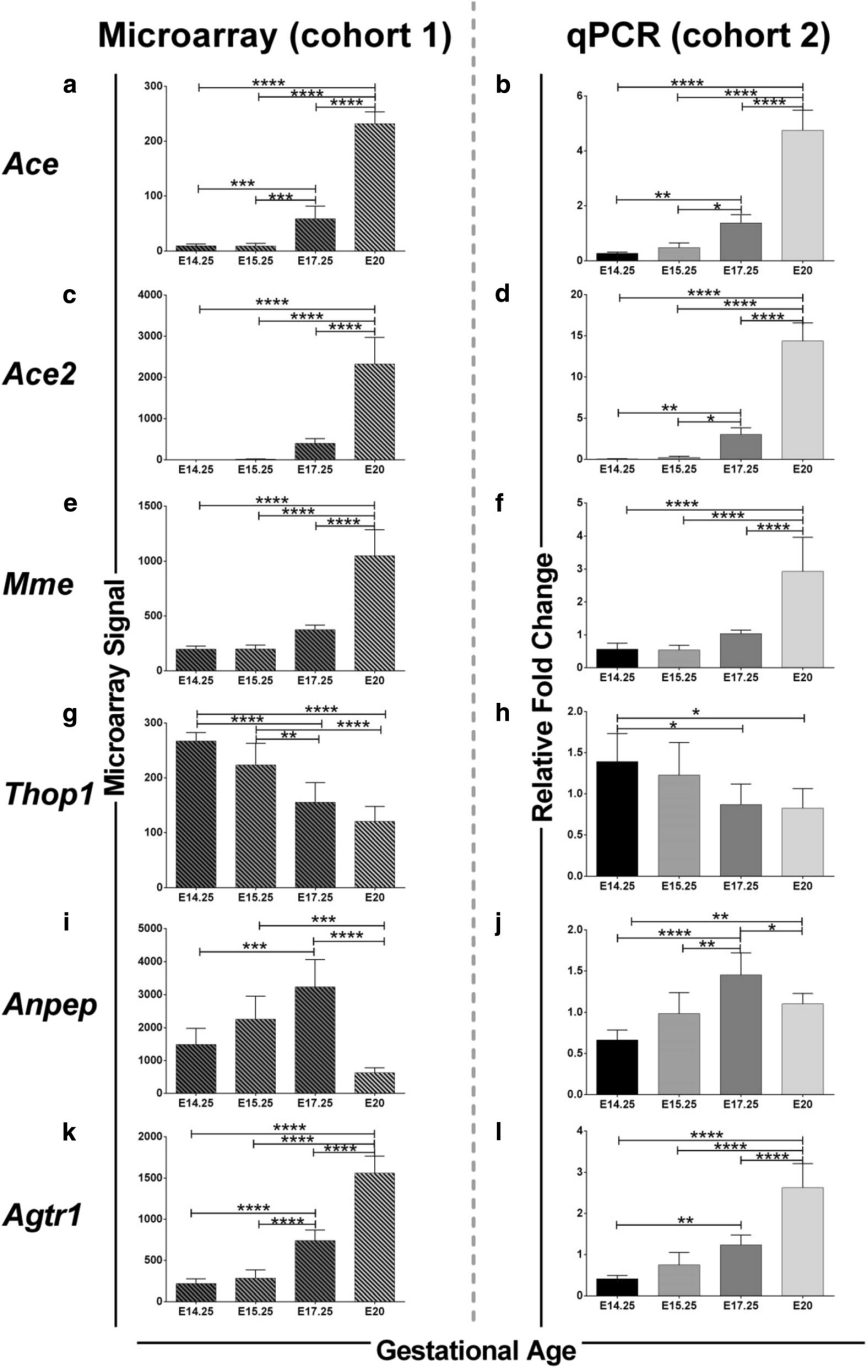
Six RAS genes that were studied for their differential expression at four gestational stages. **a** to **l** shows gene expression patterns of 6 RAS genes at E14.25, E15.25, E17.25 and E20 comparing Cohort1 (Microarray) and Cohort 2 (qPCR). Significant across the groups by 1 way ANOVA are shown in graphs as * = <0.05, ** = <0.01, *** = <0.001, and **** = <0.0001

A whole genome microarray was performed [28] to examine differential gene expression in the rat placenta at gestational ages of E14.25, E15.25, E17.25 and E20 (just prior to birth). For this study, genes of only the RAS pathways was specifically analyzed. Genes that demonstrated a >2 fold change (microarray analysis) between gestational ages were considered to be differentially expressed (Fig. 1). Six out of 17 RAS genes from the KEGG-RAS signaling pathway (*Ace, Ace2, Thop1, Mme, Anpep, and Agtr1a*) were differentially expressed (Fig. 2a, c, e, g, i, k). The other 11 genes in the pathway remained unchanged between the 4 gestational age groups (data not shown). The expression of *Ace, Ace2, Mme* and *Agtr1a* increased with gestational age whereas, *Thop1*expression was decreased with gestational age (Fig. 2). *Anpep* expression increased with gestational age and then was downregulated just prior to labour onset, hence we investigated this further at the protein level using both Western blots and immunohistochemistry.

### Validation of microarray with qPCR

Microarray results were validated by qPCR by using placenta tissue from an independent cohort of rat dams. Results for the qPCR (cohort 2) are also shown in Fig. 2 for the 6 differentially expressed RAS genes. Additional file 1: Figure S1 shows the correlation between Microarray and qPCR.

### Immunohistochemistry on ANPEP

**Fig. 3:**
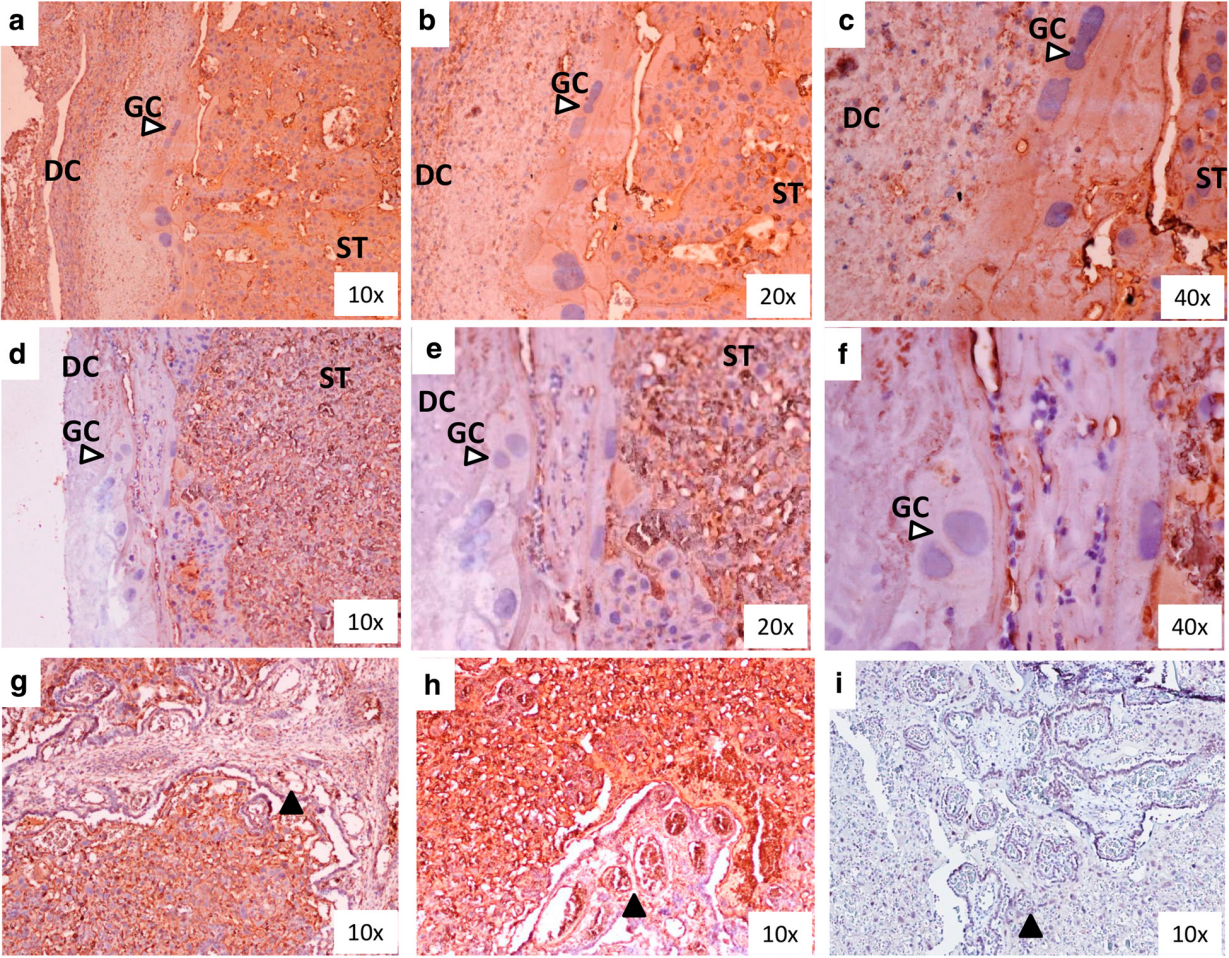
Immunohistochemistry of ANPEP(CD-13). Junctional zone displaying difference in localization of ANPEP in (panels **a** to **c**) E17.25 vs (panels **d** to **f**) E20. DC denotes decidual cells, GC giant trophoblast cells (*white arrow*), ST spongiotrophoblast layer. Labyrinth zone at E17.25 (panel **g**) and E20 (panel **h**) displaying no difference in ANPEP localization. *Black arrows* indicate blood vessles within the labyrinth zone. Panel **i** is a negative isotype (IgG) of labyrinth zone

*Anpep* gene expression increased from E14.25 to E17.25 and subsequently decreased after E17.25. Immunohistochemistry of ANPEP was performed on paraffin sections of E17.25 and E20 placentae. ANPEP was localized to both junctional and labyrinth zones (Fig. 3). In the junctional zone of E20 placentae, the decidual cells and Giant trophoblast cells had less staining compared to E17.25 placentae. Labyrinth zone localization of ANPEP remained relatively unchanged between E17.25 and E20 placentae.

### ANPEP Western blots

**Fig. 4:**
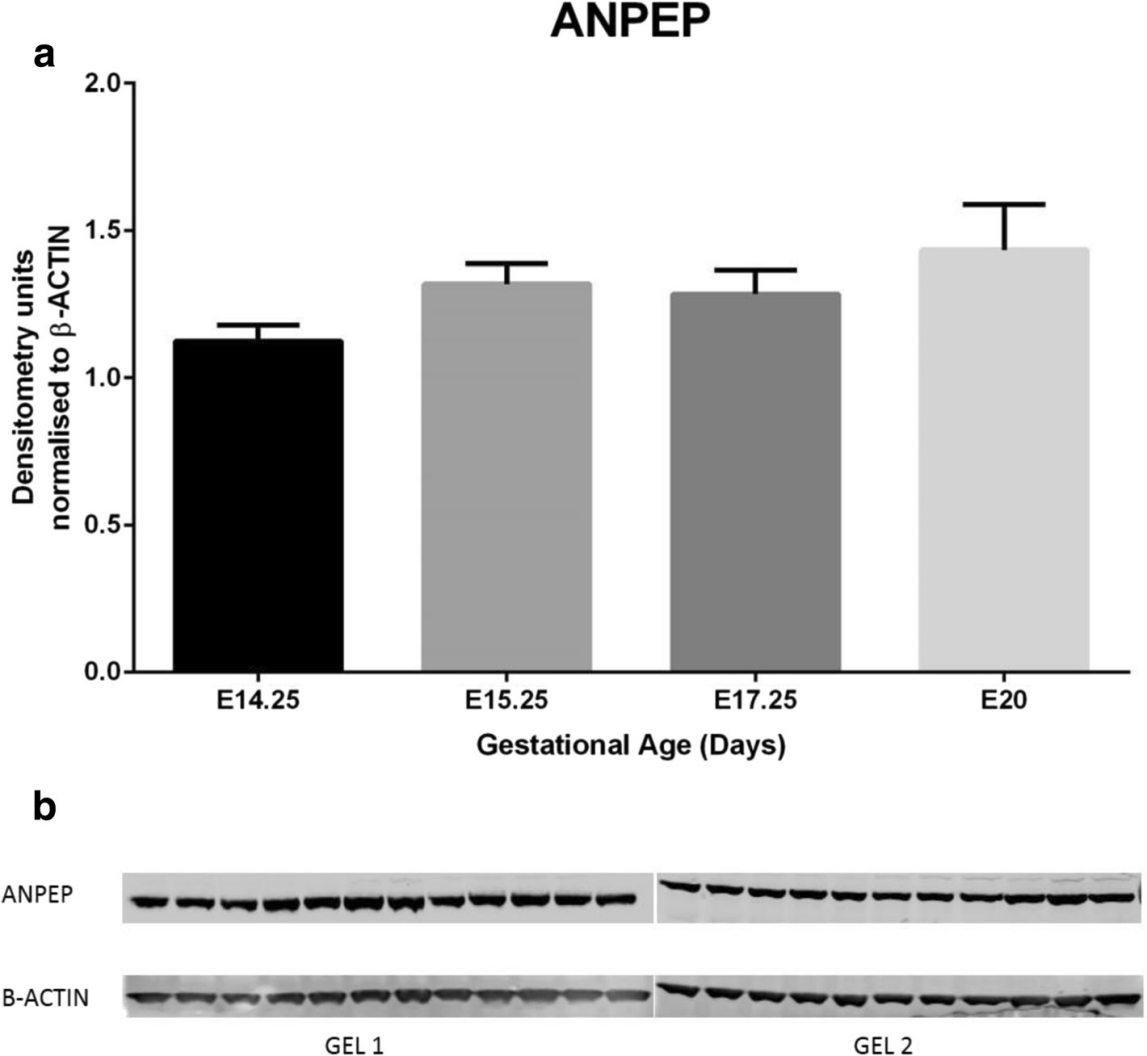
Western Blot results for ANPEP (CD-13). **a** Protein expression by Western blots displayed no significant differences across the four gestational ages by 1 way ANOVA- Multiple Comparisons. **b** Samples in order from left to right; E14.25 *n* = 6, E115.25 *n* = 5, E17.25 *n* = 6 and E20 *n* = 6

ANPEP protein expression was identified in the placentae at all four gestational ages E14.25, E15.25, E17.25 and E20. However, no significant differences in protein expression were observed within the 4 groups (Fig. 4).

## Discussion

Global RAS gene expression changes in the whole rat placenta throughout the four gestational ages E14.25, E15.25, E17.25 and E20 were studied. *Ace*, *Ace2*, *Agtr1a* (that have previously been studied in rat placenta), and three additional RAS genes *Thop1*, *Anpep* and *Mme* were significantly differentially expressed throughout mid to late gestation.

The robustness of the findings in this paper is apparent in that a similar pattern of gene expression of the 6 differentially expressed RAS genes was found in two independent rat cohorts with different techniques (microarray and qPCR, respectively) (Fig. 2). *Ace* expression was relatively low at E14.25 and E15.25, increased at E17.25 and E20. Similar results were observed for *Ace2, Mme, and Agtr1.* Consistent with previous studies, these results show that *Ace* and *Ace2* increase dramatically at late gestation, indicating that the pathway is highly activated at this stage of gestation [1, 22, 31], so these embryonic days were chosen for study. AngII has previously been shown to be increased with the advancement of pregnancy due to enhanced *Ace2* expression and activity, which correlate with elevated placental-fetal blood flow and rapid fetal growth, especially during mid and late pregnancy [1]. Dietary manipulations may have a significant role in disrupting RAS: RAS function is altered by changes in sodium intake during pregnancy [32], potentially affecting feto-maternal blood flow and hence fetal nutrient supply, fetal growth and birth weight [32]. Interestingly, another study showed that mRNA expression of *Ace2* and *Agtr1a* was significantly reduced in the labyrinth zone of Sprague Dawley rats fed a low protein diet [1]. Overall, these studies show that elevation of AngII concentration via the expression and activity of ACE and ACE2 and upregulated AT1 expression are potentially required to to increase blood flow in the placenta and therefore may ensure the adequate delivery of nutrients to the fetus, leading to optimal fetal growth.

*Mme* (membrane metallo-endopeptidase) is an enzyme that inactivates RAS by converting AngI to Angiotensin (1–7). Interestingly, MAS1 receptor gene, Mas-Related G Protein-Coupled Receptor (angiotensin 1–7 receptor) expression remained unchanged (Fig. 1). *Mme* has been studied in renal, lung and neural tissues, however, very little is known about its role in the placenta [33]. SNPs leading to truncations of the *Mme* gene and the loss of neutral endopeptidase (NEP) protein are theorised to be the cause for allo-immunisation during pregnancy [34]. Studies in rat brain and neural tissue as well as in human endometrium show that hormones can regulate *Mme* expression [35, 36]. Since MME enzyme is an important player in several tissues and in the RAS pathway, it is important to study its expression and function in placenta and pregnancy to elucidate its link to pregnancy complications.

The results of this study showed that the expression of another novel gene, *Thop1,* gradually decreases over time during pregnancy (from E14.25 to E15.25 and further decreases at E17.25) and is relatively low at E20 prior to labour onset. There is a paucity of data on *Thop1* expression and function in the placenta, however in the brain, a therapeutic potential as an anti-tumour agent has recently been reported [37, 38].

ANPEP (also known as APN) is an enzyme that selectively hydrolyses N terminal acidic amino acids such as glutamyl and aspartyl residues from peptide or substrate, and can degrade ANGII to ANGIII in the RAS pathway. It has also been previously established that ANPEP is localised to trophoblast, fetal arterioles and venules in the stem villi of the human placenta. The mRNA, protein and the activity of ANPEP is known to be higher in human placentae of pregnancies complicated with preeclampsia compared to normal pregnacy [39]. In APN-null mice, investigations on the effect of hypoxic conditions found marked reductions in formations of new blood vessels (compared to WT animals), indicating the importance of this factor in angiogenesis [24]. In the current study, *Anpep* gene expression was significantly lower at E20 (just prior to labour onset). We investigated further the protein expression of ANPEP to examine if the differences at the gene expression were detectable at the protein level. Protein expression of ANPEP (Western blot on whole placental tissue) did not significantly differ between the four groups. However, ANPEP localization by immunohistocheisty identified staing differences in E17.25 and E20 placentae, as E17.25 ANPEP localization was identified in the decidual layer, giant trophoblast cells and the spongiotrophoblast layer whereasat E20 staining was only visible in spongiotrophoblast layer (no staining in the decidual layer and giant cells). No visual differences in the localization of ANPEP was seen in labyrinth zone of the rat placentae. In future studies, differences in the protein expression by Western blot of ANPEP and other members of the RAS pathway may need to be investigated in separated junctional and labyrinth zones to fully elucidate changes to the protein expressions. Furthermore, immunohistochemical studies (such as those described in this study) may provide more extensive detail as to the cell types expressing members of the RAS pathway. The finding of these studies mayallow for more specific investigations to be performed to the role of RAS pathway members in angiogenesis as well as the overall importance of this pathway in labour. These finding may also lead to the development of functional studies ulitising knockout models in animal models (e.g. mice) of the relavent RAS factors/pathway members.

The expression of 11 RAS genes whose expression remained unchanged throughout the four gestational groups were *Agt*, *Ren*, *Ctsa*, *Cpa3*, *Nln*, *Cma1*, *Ctsg*, *Enpep*, *Mas*, *Agtr2* and *Lnpep* (data not shown). Further studies will be required to study these genes at the protein level and also comparing several regions of the rat placenta.

Many studies have indicated that RAS dysfunction is associated withpregnancy complications such as preeclampsia [25]. Normal placentataion involves trophoblast invasion of maternal decidua and remodelling of spiral arteries (replacement of smooth muscle cell layer with fibrinoid material) which causes vessels to increase in diameter allowing for increased blood flow into the placenta [40]. Abnormal placentation can lead to pregnancy complications such as preeclamsia. Preeclampsia is associated with shallow trophoblast invasion of the maternal spiral arteries in the endometrium of the uterus, leading to improper remodelling of the uterine spiral arteries [4, 31, 41]. In preeclampsia, increased sensitivity to Ang II (important in blood flow maintenance) has been documented and this phenomenon may be developed before clinical manifestations of the diesease [25]. Women with preeclampsia have been seen to have lower circulating levels of RAS factors, however, AngII levels, angiotensinogen and *Agtr1* receptor mRNA is increased in placentae of preeclamptic women [41]. Further investigations are required in normal and complicated pregnancies to fully elucidate the role of RAS in maintaining a healthy pregnancy or contributing to pregnancy complications.

## Conclusions

Adequate blood flow in the placenta is vital for the maintenance of placental function, development and fetal growth, maintenance and development. The intrauterine/placental RAS is important in pregnancy, regulating maternal-fetal blood flow and the uteroplacental blood circulation. The current study investigated the expression of members of the RAS pathway in relatively normal rat placentae at different gestational ages. Six of the 17 RAS genes were differentially expressed in the rat placenta of 4 different gestational age groups by microarray and the differernatial expressions validated by qPCR. Immunohistochemical analysis of ANPEP identified differences in localization at E20 compared to E17.25 rat placentae. These findings present the need for further comprehensive investigations of RAS genes, specific placental localizations and functional studies in normal and complicated pregnancies.

## Additional file

Additional file 1: Figure S1. Correlation plots Microarray Signal vs qPCR gene expression. A) to F) displays correlation plots for *Ace, Ace2, Mme, Thop1, Anpep, Agtr1a*. All the results were significant using Pearson Correlation. (DOCX 59 kb)
