# Supplementary material for: The rat placental renin-angiotensin system - a gestational gene expression study
Source: Reprod Biol Endocrinol. 2015 Aug 12;13:89. doi: 10.1186/s12958-015-0088-y (PMC4532142; doi:10.1186/s12958-015-0088-y)
Supplement: Additional file 1: Figure S1. — Correlation plots Microarray Signal vs qPCR gene expression. A) to F) displays correlation plots for Ace, Ace2, Mme, Thop1, Anpep, Agtr1a. All the results were significant using Pearson Correlation. (DOCX 59 kb) [file 12958_2015_88_MOESM1_ESM.docx]

**Additional file 1: Figure S1: Correlation plots Microarray Signal vs qPCR gene expression.** A) to F) displays correlation plots for *Ace, Ace2, Mme, Thop1, Anpep, Agtr1a*. All the results were significant using Pearson Correlation.

R2= 0.8066

R2= 0.9151

R2= 0.4433

R2= 0.7023

R2= 0.7383

R2= 0.2475
